# In Vitro Antibacterial Activity of Manuka (*Leptospermum scoparium* J.R. et G. Forst) and winter Savory (*Satureja montana* L.) Essential Oils and Their Blends against Pathogenic *E. coli* Isolates from Pigs

**DOI:** 10.3390/ani10122202

**Published:** 2020-11-24

**Authors:** Filippo Fratini, Mario Forzan, Barbara Turchi, Simone Mancini, Giuseppe Alcamo, Francesca Pedonese, Luisa Pistelli, Basma Najar, Maurizio Mazzei

**Affiliations:** 1 Department of Veterinary Sciences, University of Pisa, Viale delle Piagge 2, 56124 Pisa, Italy; mario.forzan@unipi.it (M.F.); barbara.turchi@unipi.it (B.T.); simone.mancini@unipi.it (S.M.); giuseppe.alcamo@virgilio.it (G.A.); francesca.pedonese@unipi.it (F.P.); maurizio.mazzei@unipi.it (M.M.); 2 Department of Pharmacy, University of Pisa, Via Bonanno 6, 56126 Pisa, Italy; luisa.pistelli@unipi.it (L.P.); basmanajar@hotmail.fr (B.N.)

**Keywords:** swine neonatal diarrhoea, swine post-weaning diarrhoea, oedema disease, ETEC, EPEC, synergy

## Abstract

**Simple Summary:**

Neonatal diarrhoea (ND), post-weaning diarrhoea (PWD) and oedema disease (OD) are particularly important in terms of economic losses in pig farming worldwide. Aetiological agents of these diseases belong to *Escherichia coli*, in particular to a few serogroups of enterotoxigenic *Escherichia coli* (ETEC) and enteropathogenic *Escherichia coli* (EPEC). The necessity for a reduction in antibiotic use, related to the growing antibiotic resistance phenomenon, encourages research in the study of alternative antibacterial substances as antibacterial tools. Essential oils could represent a valid solution.

**Abstract:**

Neonatal diarrhoea (ND), post-weaning diarrhoea (PWD) and oedema disease (OD) are among the most important diseases affecting pig farming due to economic losses. Among the main aetiological agents, strains of *Escherichia coli* are identified as the major responsible pathogens involved. Several strategies have been put in place to prevent these infections and, today, research is increasingly studying alternative methods to antibiotics to reduce the antibiotic resistance phenomenon. Essential oils (EOs) are among the alternative tools that are being investigated. In this study, the in vitro effectiveness of winter savory and manuka essential oils and their mixtures in different proportions against strains of *E. coli* isolated from episodes of disease in pigs was evaluated. The EOs alone demonstrated slight antibacterial effectiveness, whereas the blends, by virtue of their synergistic action, showed remarkable activity, especially the 70%–30% winter savory–manuka blend, showing itself as a potential tool for prevention and therapy.

## 1. Introduction

The presence of different *Escherichia coli* strains in the swine intestinal tract must be considered a normal condition; in fact, many of these strains live in a symbiotic way with the rest of the faecal flora and represent a substantial part of the swine intestinal microbiota [1]. Nevertheless, if they acquire genes responsible for virulence, they may become causative agents of various forms of illness [1]. In light of the above, several *E. coli* strains have been recognized as important aetiological agents of several diseases in pigs worldwide, i.e., neonatal septicaemia, neonatal diarrhoea (ND), post-weaning diarrhoea (PWD), oedema disease (OD), cystitis, septicaemia, polyserositis, coliform mastitis and urinary tract infections. Moreover, these strains are able to colonise existing lesions elsewhere in other organs [2]. Among the most important illnesses for the economic consequences occurring in pig farms are undoubtedly ND, PWD and OD [3]. ND is a worldwide spreading disease in pigs and responsible for economic losses due to the significant increase in both morbidity and mortality of piglets [3]. In the same way, PWD, also known as post-weaning enteric colibacillosis, is widespread globally and furthermore represents an important cause of death in weaned pigs. However, enteric infection may also occur only with diarrhoea, affecting piglets during the first week after weaning and resulting in a reduction in weight gain [4]. PWD is mainly caused by enteropathogenic *E. coli* (EPEC) strains, although enterotoxigenic *E. coli* (ETEC) strains are very often involved [5,6]. Porcine pathogenic *E. coli* involved in PWD typically belong to serogroups O:8, O:138, O:139, O:141, O:147, O:157 and O:149, the latter being the predominant serogroup in most countries. However, O serogroup and virulence gene patterns vary considerably from region to region and over time [7]. OD represents an important cause of mortality in pig farming in Europe. Among the ETEC strains, the serotypes involved in the aetiology of this pathology appear to be O:138, O:139 and O:141, responsible for the production of toxins that reach the bloodstream, damaging the blood vessel walls in different tissues and organs [8]. The main clinical signs of the disease are abundant oedema of the stomach and mesocolon, often caused by strains able to produce a massive amount of adhesion factors and, after colonising the intestines, different toxins [9]. The high incidence of these diseases combined with the need for a reduction in antibiotic use, in the light of the growing antibiotic resistance phenomenon, stimulated researchers to study alternative molecules to be used in illness prevention and in therapy [10]. During the last few years, essential oils (EOs) have been widely investigated overall for their activity as growth promoter due to their antioxidative properties [11]. Unfortunately, so far, only few data are available in the literature about their antibacterial activity against causative agents of swine diseases [12]. Lately, the interest of the scientific world in EOs has increased due to their potential employment as an effective alternative treatment to several animal diseases, although most research has been carried out by in vitro assays [13,14].

Among the EOs tested for their effectiveness against many microorganisms responsible for pathologies in humans and animals, the most effective were those obtained from plants belonging to the Lamiaceae family due to their abundance of bioactive monoterpenes [11,15,16]. Although several studies have focused on the activity of aromatic plants belonging to this family and the antibacterial properties of their EOs, such as oregano, marjoram, sage, rosemary and other EOs, few data are available about winter savory (*Satureja montana* L.) and its derived EO [17]. Winter savory is a multiannual semi-shrubbery plant that grows in several Mediterranean regions, able to develop a great number of morphological and physiological adaptations [18]. The essential oil obtained from various species of *Satureja* have highlighted different antibacterial biological properties [13,19,20]. The main compounds in winter savory EOs are carvacrol, gamma-terpinene and p-cymene, responsible for antibacterial activity [19]. In addition, manuka (*Leptospermum scoparium* J.R. et G. Forst) is a small tree or more often a shrub belonging to the Myrtaceae family, widespread in Australia and New Zealand [21]. Although little is known about the therapeutical use of the plant, its essential oil is effective against a wide range of microorganisms, mostly Gram-positive bacteria, including antibiotic-resistant strains [22]. The most important manuka EO compounds are leptospermone, *iso*-leptospermone and flavesone, the main triketone constituents [21,22,23].

In order to obtain a potential natural antimicrobial alternative product which could be used against pathogenic bacteria in pig farming, this investigation aimed to determine the in vitro antibacterial activity of manuka and winter savory EOs both singularly and in combination against three bacterial strains isolated from episodes of ND, PWD and OD.

## 2. Materials and Methods

### 2.1. Bacterial Strains

Three *E. coli* wild strains, belonging to the strain collection of the Department of Veterinary Sciences of University of Pisa (Pisa, Italy) and stored at −80 °C in a glycerol suspension, were employed in this research. The three bacterial strains were isolated from episodes of ND, PWD and OD, previously identified and serotyped as O:8, O:149 and O:139 [24]. Strains were also previously screened and resulted positive for the presence of genes codifying for some of the main virulence factors such as stable toxin a (STa) and labile toxin (LT I) [25]. Moreover, the antibiotic resistance profile of the isolates, obtained using the disc-diffusion method according to the Kirby–Bauer test and interpreted according to European Committee on Antimicrobial Susceptibility Testing (EUCAST) [26], is reported in Table 1.

### 2.2. Essential Oils and Blends Employed in the Trials

Essential oils (EOs) of *Satureja montana* L. (*Sm*) and *Leptospermum scoparium* J.R. et G. Forst (*Ls*) were purchased from FLORA^®^ s.r.l. (Lorenzana, Pisa, Italy; lot numbers 142012 and 151939, respectively). According to the certificates provided by the company, *Sm* and *Ls* originated from France and New Zealand, respectively, and both EOs were extracted by steam distillation from the entire flowering plant for *Sm* EO and from the leaves for *Ls* EO. In order to evaluate a potential combined action of the EOs, three mixtures were prepared as follows: 50% *Sm* + 50% *Ls*; 30% *Sm* + 70% *Ls*; 70% *Sm* + 30% *Ls*. EOs were stored at 4 ± 2 °C in the dark and were subjected to microbiological analysis for quality control before their employment in the tests.

### 2.3. Gas Chromatography–Electron Impact Mass Spectrometry

The gas chromatography–electron impact mass spectrometry (GC–EIMS) analysis was carried out according to the instructions reported by Pistelli et al. [27] in the laboratory of the Department of Pharmacy (University of Pisa).

### 2.4. Determination of Minimum Inhibitory Concentration (MIC) and Minimum Bactericidal Concentration (MBC) of EOs Alone and in Blends

The Eos’ MIC values for each strain were determined using the twofold serial microdilution method according to the Wiegand et al. [28] protocol with some modifications previously reported in Fratini et al. [23]. Both MIC and MBC results were expressed as *v*/*v*. The same procedure for the determination of MIC and MBC values for individual EOs was also applied to the blends.

## 3. Results and Discussion

The composition of single EOs and blends is reported in Table 2. The main compounds in *Sm* were carvacrol (45.4%), p-cymene (10.3%) and thymol (7.0%), and the most representative in *Ls* were cis-calamenene (21.4%), leptospermone (18.3%) and flavesone (6.9%). As regards the compounds’ concentration in the Sm and Ls Eos, it is interesting to note that Ls and Sm compositions were slightly different from what was reported previously [29]. These little differences may be due to the degradation of components during the refrigerated storage. Concerning the EO blends, the main compounds were affected, as expected, by the chemical composition of the single EOs. In detail, for the 70%–30% Sm/Ls blend, the three main components were carvacrol (29.0%), leptospermone (7.0%) and p-cymene (6.6%); for the 50%–50% Sm/Ls blend, carvacrol was present at a percentage of 22.1%, followed by leptospermone at 10.5% and cis-calamenene at 6.5%; finally, the 30%–70% Sm/Ls blend showed to be mainly constituted by carvacrol (13.6%), leptospermone (12.9%) and cis-calamenene (8.9%).

All the data obtained in triplicate are reported in Table 3. The MIC and MBC values obtained for each strain with both the single EOs showed a weak antibacterial activity. Sm EO showed mode MIC values of 1:64 against each of the *E. coli* strains, whereas Ls EO showed low values of MIC equal to 1:32. The strong action of Ls EO was attributed to its high amount of leptospermone (18.3%), a β-triketone known, like its derivatives, for its great antimicrobial power [30,31].

The best values of inhibition were obtained with the blends of the two oils; in particular, the most encouraging MIC values were obtained with the 70%–30% Sm/Ls blend for all the tested strains, as reported in Table 3. In detail, with the 70%–30% Sm/Ls blend, the MIC mode value for the strain O:149 is 1:512, and for the strains O:139 and O:8 this value is 1:256; with the 50–50% Sm/Ls blend, the MIC mode value is 1:256 for the strain O:149, while this value is 1:128 for the other two strains. An MIC mode value equal to 1:128 was also obtained with the 30–70% Sm/Ls blend for all the bacterial strains. Although the 70%–30% Sm/Ls blend is the most active, it seems evident that the mixtures have a higher inhibitory activity than the oils used individually, confirming the greater effectiveness of synergistic action. These mixtures were characterised by the presence of two compounds known by their antibacterial activity, namely carvacrol and leptospermone. The 70%–30% Sm/Ls mixture was the most effective, probably due to the presence of these two compounds at amounts of 29.0% and 7.0%, respectively. The presence of other compounds such as p-cymene and thymol can enhance this efficacy since these two constituents are also well documented for their antibacterial activity [32,33]. All these hypotheses can be considered valid, taking also into consideration the potential synergism between these constituents [34,35]. Concerning the MBC values, it is interesting to observe how, in the case of Sm EO, these are superimposable to the MIC values in the three replicates for all three bacterial strains, demonstrating an inhibitory and bactericidal effect at the same concentrations. However, in the case of Ls EO, the MBC values are often offset by one step, between 1:16 and 1:32. Regarding MBC mode values for the blends, the following results were obtained: concerning the 50%–50% Sm/Ls blend for the strain O:149, we observe a mode MBC value of 1:256 equal to the mode MIC value for the same strain; for the strains O:139 and O:8, we note MBC mode values of 1:64; with respect to the 30%–70% Sm/Ls blend, we observe a mode MBC value of 1:128 for the strains O:149 and O:8, and one of 1:64 for the strain O:139; finally, with respect to the 70%–30% Sm/Ls blend, the MBC mode value for the strains O:149 and O:8 is 1:256, while for the strain O:139 it is 1:128.

Analysing the obtained results, the synergistic action highlighted in vitro by the mixtures of essential oils compared to the oils used individually against all three bacterial strains is noticeable. The greater effectiveness of essential oil combinations with respect to single oils against *E. coli* strains is however supported by several works in the literature [13,36,37,38]. Indeed, Fu et al. [36] reported a synergistic effect of clove and rosemary essential oil blends, as also reported by de Medeiro Barbosa et al. [38] for mixtures of rosemary and oregano oils tested via the fractional inhibitory concentration (FIC). The powerful synergistic effect found in blends compared to the oils tested alone could be attributed to some individual and less abundant compounds with a remarkable antibacterial activity, such as p-cymene [13], γ-terpinene [39], isothymol methyl ether [40] and β-caryophyllene [41] mainly represented in Sm EO, and other molecules such as α-cubebene, α-copaene, α-selinene, β-selinene [42] and flavesone [43] mainly represented in Ls EO.

Despite the results obtained in this investigation, the main problem in the use of each plant extract remains the instability of its composition, influenced by numerous factors such as climate, season, harvesting methods, etc. To overcome these drawbacks, it might seem useful to select the extracts, standardise them and verify their synergistic action, but in doing so the actions of the minority components present in the phytocomplex would be lost. However, due to the promising results obtained in in vitro tests, it would be desirable to set up experiments to evaluate the effect of these blends on porcine intestinal epithelial cells and then move on to a subsequent step where the same mixtures in episodes of ND, PWD and OD are tested. Finally, recent acquisitions of micro-encapsulation and the development of nanotechnologies could certainly represent the tools for the EOs and their blends to reach the pig intestinal sites in order to express their effectiveness against many pathologies in pig farming.

## 4. Conclusions

Essential oils (EOs) are one of the most promising alternative tools to control livestock diseases. In vitro effectiveness of winter savory and manuka essential oils was enhanced by their blends against *E. coli* isolated from pigs’ diseases, such as neonatal diarrhoea, post-weaning diarrhoea and oedema disease. These essential oil blends could be taken into account as potential antibacterial treatments for prevention and therapy, also in the light of increasing antibiotic resistance concern.

## Figures and Tables

**Table 1:** Phenotypic antibiotic resistance profile of the three isolates according to EUCAST [26].

Antibiotic ^1^	*E. coli* O:149	*E. coli* O:139	*E. coli* O:8
AMC	S	S	S
AMP	S	S	S
ATM	S	S	R
C	S	S	S
CTX	S	S	R
ENR	S	S	S
FOX	S	S	R
GEN	S	S	S
IPM	S	S	S
KF	R	R	R
STR	R	R	R
SXT	S	S	S
TET	S	R	S

^1^ Antibiotic abbreviations: amoxicillin–clavulanic acid (AMC), ampicillin (AMP), aztreonam (ATM), chloramphenicol (C), cefotaxime (CTX), enrofloxacin (ENR), cefoxitin (FOX), gentamicin (GEN), imipenem (IPM), cephalothin (KF), streptomycin (STR), trimethoprim–sulfamethoxazole (SXT), tetracycline (TET).

**Table 2:** Essential oil (EO) composition of winter savory (*Sm*), manuka (*Ls*) and the three *Sm–Ls* blends (70% *Sm* + 30% *Ls*; 50% *Sm* + 50% *Ls*; 30% *Sm* + 70% *Ls*).

Compounds	Class	LRI ^1^	*Sm*	*Ls*	*Sm–Ls*_70:30_	*Sm–Ls*_50:50_	*Sm–Ls*_30:70_
α-pinene	mh	940	0.9	0.9	0.9	0.8	0.9
camphene	mh	954	0.5	-	0.3	0.2	0.2
sabinene	mh	975	0.5	-	0.3	0.2	0.2
1-octen-3-ol	nt	979	0.6	-	0.4	0.3	0.2
myrcene	mh	991	1.3	0.3	0.9	0.7	0.6
α-terpinene	mh	1017	1.3	-	0.9	0.5	0.4
*p*-cymene	mh	1025	10.3	0.3	6.6	4.5	3.1
limonene	mh	1029	3.0	-	2.0	1.4	0.5
1,8-cineol	om	1031	-	0.3	-	-	0.6
γ-terpinene	mh	1060	6.2	-	4.3	3.2	2.1
linalool	om	1097	1.5	-	1.0	0.6	0.4
borenol	om	1169	3.0	-	1.9	1.2	0.8
4-terpineol	om	1177	1.6	-	1.0	0.6	0.4
α-terpineol	om	1189	1.4	-	0.9	0.5	0.2
isothymol methyl ether	om	1244	5.2	-	3.3	2.3	1.6
thymol	om	1290	7.0	-	4.4	3.1	2.1
carvacrol	om	1299	45.4	-	29.0	22.1	13.6
α-cubebene	sh	1351	-	3.0	1.6	1.6	2.1
α-copaene	sh	1377	0.3	4.6	1.9	2.5	3.2
β-elemene	sh	1391	-	0.6	0.2	0.3	0.4
α-gurjunene	sh	1410	0.1	1.2	0.4	0.5	0.8
β-caryophyllene	sh	1419	3.5	2.2	2.8	2.6	2.4
α-guaiene	sh	1440	0.3	2.0	0.8	1.1	1.5
*cis*-muurola-3.5-diene	sh	1450	-	1.4	0.8	1.2	1.7
*allo*-aromadendrene	sh	1460	-	0.8	0.3	0.4	0.6
*trans*-cadina-1(6),4-diene	sh	1477	-	2.2	1.1	1.4	2.2
γ-muurolene	sh	1480	0.2	1.4	0.6	0.8	0.9
β-selinene	sh	1490	0.2	4.8	1.9	2.7	3.4
*cis*-β-guaiene	sh	1493	-	0.5	0.3	-	0.5
α-selinene	sh	1498	-	4.8	2.0	2.9	3.5
α-muurolene	sh	1499	-	0.9	-	-	-
*trans*-β-guaiene	sh	1503	-	-	0.4	0.5	0.6
(*E,E*)-α-farnesene	sh	1506	-	0.5	-	-	0.7
β-bisabolene	sh	1509	0.8	-	0.7	0.7	-
*trans*-γ-cadinene	sh	1514	0.2	1.1	0.5	0.7	0.8
δ-cadinene	sh	1523	0.6	-	3.3	4.1	4.5
*trans*-cadina-1(2),4-diene	sh	1535	-	3.0	1.5	2.2	2.6
*cis*-calamenene	sh	1540	-	21.4	3.3	6.5	8.9
flavesone	nt	1547	-	6.9	2.5	3.9	4.7
spathuneol	os	1578	-	0.8	0.3	0.4	0.6
caryophyllene oxide	os	1583	0.8	0.8	1.1	1.2	1.3
globulol	os	1585	-	0.7	-	0.1	-
viridiflorol	os	1593	-	0.5	0.2	0.2	0.4
*iso*-leptospermone	os	1621	-	6.5	2.0	3.3	4.4
leptospermone	os	1629	-	18.3	7.0	10.5	12.9
T-cadinol	os	1642	-	1.7	0.9	1.2	1.0
selin-11-en-4-α-ol	os	1660	-	0.9	0.2	0.3	0.6
occidentalol acetate	os	1682	-	0.5	-	0.3	0.4
**Class of Compounds**			***Sm***	***Ls***	***Sm–Ls*_70:30_**	***Sm–Ls*_50:50_**	***Sm–Ls*_30:70_**
Monoterpene hydrocarbon (mh)			24.0	1.5	16.2	11.5	8.0
Oxygenated monoterpenes (om)			65.1	0.3	41.5	30.4	19.7
Sesquiterpene hydrocarbon (sh)			6.2	56.4	24.4	32.7	41.3
Oxygenated sesquiterpenes (os)			0.8	30.7	11.7	17.5	21.6
Non-terpenes (nt)			0.6	6.9	2.9	4.2	4.9
Total identified			96.7	95.8	96.7	96.7	95.5

^1^ LRI: linear retention indices on DB-5 column. Compound concentrations expressed as percentage of total identified amount.

**Table 3:** Mode of minimum inhibitory concentration (MIC) and minimum bactericidal concentration (MBC) values of winter savory (*Sm*) and manuka (*Ls*) essential oils (EOs) against *Escherichia coli* wild strains O:149, O:139 and O:8.

	MIC				
*Strains*	*Sm*	*Ls*	*Sm–Ls*_70:30_	*Sm–Ls*_50:50_	*Sm–Ls*_30:70_
*E. coli* O:149	1:64	1:32	1:512	1:256	1:128
*E. coli* O:139	1:64	1:32	1:256	1:128	1:128
*E. coli* O:8	1:64	1:32	1:256	1:128	1:128
	**MBC**				
***Strains***	***Sm***	***Ls***	***Sm–Ls*_70:30_**	***Sm–Ls*_50:50_**	***Sm–Ls*_30:70_**
*E. coli* O:149	1:64	1:16	1:256	1:256	1:128
*E. coli* O:139	1:64	1:16	1:128	1:64	1:64
*E. coli* O:8	1:64	1:32	1:256	1:64	1:128
